# Empathy competence and future specialty among medical residents in Japan: a nationwide cross-sectional study

**DOI:** 10.1038/s41598-023-41011-w

**Published:** 2023-08-23

**Authors:** Takashi Watari, Nathan Houchens, Yuji Nishizaki, Koshi Kataoka, Tomoe Otsuka, Yasuhisa Nakano, Kota Sakaguchi, Yoshihiko Shiraishi, Kohta Katayama, Hitomi Kataoka, Yasuharu Tokuda

**Affiliations:** 1 General Medicine Center, Shimane University Hospital, Izumo, Shimane Japan; 2 Medicine Service, VA Ann Arbor Healthcare System, Ann Arbor, MI USA; 3 Department of Medicine, University of Michigan Medical School, Ann Arbor, MI USA; 4 Medical Technology Innovation Center, Juntendo University, Bunkyo-ku, Tokyo, Japan; 5 Faculty of Medicine, Shimane University, Izumo, Shimane Japan; 6 Department of Internal Medicine, St. Marianna University School of Medicine, Kawasaki, Kanagawa Japan; 7 Diversity and Inclusion Center, Okayama University Hospital, Okayama, Japan; 8 Muribushi Okinawa Project for Teaching Hospitals, Okinawa, Japan

**Keywords:** Health care, Medical ethics

## Abstract

Empathy is essential for physicians to provide patient-centered care. Nevertheless, the degree to which empathy varies among medical residents based on their desired future specialty remains undetermined. This nationwide cross-sectional study compared empathy levels (Jefferson Scale of Empathy, JSE) of 824 year one and two postgraduate residents in Japan by intended medical specialty, individual characteristics, and training and working environment characteristics. Empathy levels were compared with applicants for general medicine, which emphasizes patient-centeredness. The highest mean JSE and the highest percentage of women residents were observed in general medicine (M = 109.74; SD = 14.04), followed by dermatology (M = 106.64; SD = 16.90), obstetrics and gynecology (M = 106.48; SD = 14.31), and pediatrics (106.02; SD 12.18). Residents interested in procedure-centered departments (e.g. ophthalmology, orthopedics) garnered lower JSE scores. Multivariate regression revealed that future general medicine candidates achieved the highest JSE scores ($\beta$ = 6.68, 95% CI 2.39–10.9, p = 0.002). Women achieved significantly higher JSE scores than men ($\beta$ = 2.42, 95% CI 0.11–4.73, p = 0.041). The results have implications for empathy training and postgraduate education strategy in different clinical specialties.

## Introduction

The ability to demonstrate empathy is critically important in the physician–patient relationship. Empathic communication allows physicians to establish and maintain a connection with patients and more fully understand them^1–3^. Hojat et al. proposed the following definition of empathy in the context of patient care: “Empathy is a predominantly cognitive (rather than emotional) attribute that involves an understanding (rather than feeling) of the experiences, concerns, and perspectives of the patient, combined with a capacity to communicate this understanding and an intention to help”^4^.

Using the Jefferson Scale of Empathy (JSE), for which validity and reliability have been established across multiple populations and cultural contexts^1,5–8^, numerous prior studies have demonstrated the importance of empathy for both patients and providers in various clinical and medical education settings. For example, higher empathy in healthcare providers has been associated with fewer cardiovascular events among patients under their care^9^ and better management outcomes for such conditions as diabetes mellitus, dyslipidemia^10^, and obesity^11^. Furthermore, enhanced empathy has been correlated with a shorter duration of common cold symptoms^12^. In addition to improved clinical health outcomes in patients, studies have reported that higher empathy may lead to reduced medical litigation^13^ and physician burnout^14^, and may increases physician happiness and well-being^15^.

Many variables appear to influence levels of empathy among healthcare providers. Among all medical professionals, women demonstrate higher empathy scores than men^7,16–18^, which is particularly notable given that empathy is negatively affected (i.e. reduced) over the medical training period^19,20^. In particular, levels of empathy among healthcare providers may be higher in departments emphasizing patient-centeredness. For instance, a US study found that primary care physicians were the most empathic^3,21^. Prior studies have reported that, among medical students, a high degree of empathy is strongly associated with interest in primary-care-related fields generally and general medicine in particular^22,23^.

Conversely, various studies across countries have demonstrated that empathy is lower among procedure-oriented physicians, such as surgeons^21–24^. However, relative levels of empathy among physicians in each specialty may also be influenced by training and professional practice experience, as well as by the external environment (e.g. litigation, conflicts with patients). Beyond the role of sex^21^, the relationship between the career path of a resident and empathy levels may be related to such factors as their respective training environments, working conditions, and the presence of a mentor.

However, to the best of our knowledge, there is limited research exploring the correlation between residents’ empathy and their intended specialty before specialty training^22–24^. General medicine, characterized by practitioners with notable empathic capacities, has a brief history in Japan, having been officially recognized as a specialty only in 2018^25,26^. Because of a lack of scientific evidence, it is necessary to clarify the extent to which empathy is found among trainees who are planning to join different future specialties in Japan, a country in which the medical system, payment structure, physician salary, and cultural backgrounds are distinct from the Western cultures in which previous studies were conducted^3,16,22–24^.

The primary objective of this study is to ascertain whether empathy levels among medical residents in Japan differ according to their anticipated specialty choice. Thus, we compared empathy levels based on the characteristics of residents, training environments, and working environments. The second objective was to explore empathy levels among applicants for general medicine in Japan, a specialty that emphasizes the principle of patient-centeredness.

## Methods

### Participants and data collection

This was a nationwide, cross-sectional study. Between January 18, 2021, and March 31, 2021, we used an electronic survey request to enroll first-year (PGY-1) and second-year (PGY-2) postgraduate residents in Japan who had recently completed the General Medicine In-Training Examination (GM-ITE). The study design followed the STROBE guidelines.

### The Japanese postgraduate clinical training system

In the Japanese training system, after graduating from 6 years of medical school, residents must complete 2 years of mandatory rotational training before proceeding to a specialist medical program over 24 months. The seven rotational trainings consist of internal medicine, surgery, rural community medicine, obstetrics and gynecology, pediatrics, psychiatry, and anesthesiology, along with some elective programs^27^. During the first two postgraduate years, trainees, who are called “residents” or “junior residents,” use this period to choose their future specialties. Subsequently, they usually train in one of 19 major specialties in the third year after graduation. There is no competition (i.e. participating in matches against specialty training programs), and most residents are free to proceed to later training according to their medical interests^28,29^.

### General medicine in-training examination

The GM-ITE is a multiple-choice, 80-question clinical examination that assesses general medical knowledge and its application in accordance with the core curriculum of the training program of the Ministry of Health, Labor, and Welfare of Japan^30^. More than 50% of all resident physicians take this exam annually^30,31^. The examination scope comprehensively covers the most frequent topics to be mastered during essential training regarding internal medicine, surgery, anesthesiology, emergency medicine, pediatrics, psychiatry, obstetrics and gynecology, and others^30^. Upon completion of the examination, candidates are provided feedback based on their relative scores and detailed explanations for each question.

### Data collection

After the culmination of the GM-ITE, the research participants were asked to further contribute voluntarily. Consent was procured prior to the execution of a self-administered electronic questionnaire, encompassing queries pertaining to the training hospital milieu, pertinent information about the resident, and their chosen future specializations^31^. Additional questions included the number of emergency room shifts per month, duty shifts per month, hours worked per week, hours of self-study time, and the average number of inpatients in their care. Information on each hospital’s basic characteristics was obtained from the Resident Electronic Information System website and Foundation for Medical Training. The classification of hospital characteristics into urban and regional cities was based on previous studies^31^.

### Exclusion criteria

**Figure 1:**
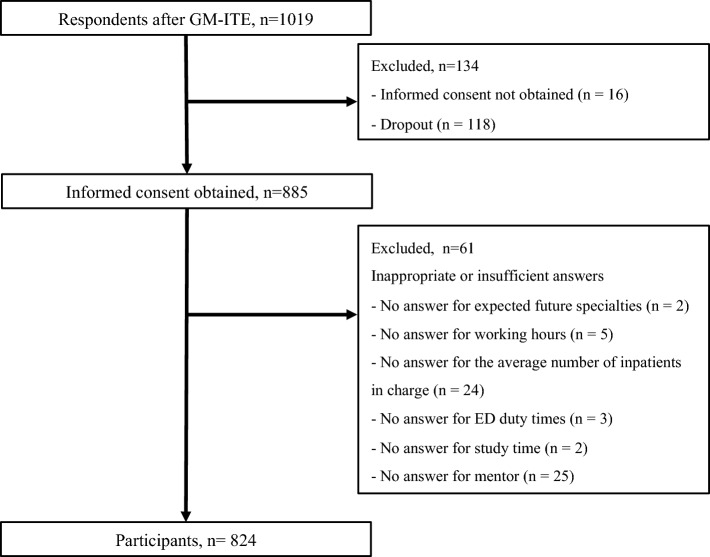
Flow diagram for study participants.

Among all participants (1019) who remained after completing the GM-ITE, 134 were excluded (118 did not undertake the subsequent survey; 16 did not consent). After another 61 respondents were excluded for other reasons (Fig. 1), a total of 824 participants were included in the analysis.

### Statistical analysis

The JSE total score was used as the primary outcome (independent variable). To assess the internal consistency of reliability of the JSE, we calculated Cronbach’s alpha; to estimate effect size, we calculated Cohen’s d from the *t* test differences between the two groups. Standard descriptive statistics were used to calculate the number, percentage, mean, median, and interquartile range (IQR) of each variable in the dataset. The chi-square test or Fisher’s exact test was used to compare categorical data. Additionally, multivariate linear regression analysis was performed to examine factors associated with total scores on the JSE, which were adjusted based on clinical relevance and previous studies. We also examined variance inflation factors (VIF) to confirm the absence of multicollinearity. Finally, sensitivity analysis was performed by incorporating random effects (i.e. hospital-level variables and resident-level variables) in the multiple regression analysis. All tests were two-tailed, and statistical significance was set at p < 0.05. All analyses were performed using STATA (Stata Corp. 2015, Stata 17 Base Reference Manual).

### Ethical considerations

Ethical approval for this research was obtained from the Ethical Review Committee of Japan Organization of Advancing Medical Education Program, JAMEP, No. 21-7. All participants provided written informed consent before participating in the study in accordance with the Declaration of Helsinki.

## Results

A total of 824 residents who consented to participate in the study were included in the analysis (PGY-1 = 364, PGY-2 = 460). Cronbach’s alpha was 0.85. Table 1 shows the characteristics of the hospital training sites and residents. Most (81.4%) were trained in community hospitals, 71% were trained in rural areas, and more than 90% of training hospitals had more than 300 beds (mean [M] 552.36, SD 218.8). At the resident level, 28.0% of respondents were women, 55.8% PGY-2, and approximately 70% were on duty in the emergency department 3–5 times monthly, with the most common response of being in charge of 5–9 patients at a time. Sixty-eight percent of residents reported having at least one mentor. The mean GM-ITE score was 46.29 out of a total possible score of 80, and the mean JSE score was 103.61 (SD 15.0%). Women’s JSE scores were significantly higher than those of men (women M = 105.66, SD 14.08; men M = 102.81, SD 15.25; p = 0.01).

**Table 1:** Background factors and resident characteristics.

	ALL, n = 824 (% or SD)
Hospital-level variables	
Hospital type (%)	
University	98 (11.9%)
University branch	55 (6.7%)
Community	671 (81.4%)
Hospital location (%)	
Urban	239 (29.0%)
Rural	585 (71.0%)
Number of beds	Mean 552.36 (SD 218.8)
Resident-level variables	
Sex (%)	
Men	593 (72.0%)
Women	231 (28.0%)
PGY (%)	
PGY-1	364 (44.2%)
PGY-2	460 (55.8%)
ED duties per month (%)	
None	37 (4.5%)
1–2	130 (15.8%)
3–5	587 (71.2%)
6 or more	70 (8.5%)
Average number of inpatients in charge (%)	
0–4	220 (26.7%)
5–9	497 (60.3%)
10–14	78 (9.5%)
15 or more	29 (3.5%)
Resident duty hours per week (%)	
59 or fewer	298 (36.2%)
60–79	333 (40.4%)
80 or more	193 (23.4%)
Have mentor(s)?	
Yes	561 (68.1%)
No	263 (31.9%)
Jefferson scale of empathy	103.61 (15.0%)
GM-ITE score	46.29 (7.3%)

*PGY* postgraduate years, *GM*-*ITE* general medicine in-training examination, *ED* emergency department.

Table 2 shows the average JSE score and the percentage of women residents in each department of interest (19 primary areas and others not yet decided). Internal medicine, which encompasses many subspecialties, such as cardiovascular, respiratory, gastrointestinal, and collagen disease, had the highest number, with 325 residents (JSE M = 104.60, SD = 14.56), accounting for 39.4% of all residents. Surgery had the next highest number of residents at 90, which accounted for 10.9% of all residents (JSE M = 103.39, SD = 15.32). The department with the highest empathy was general medicine (JSE M = 109.74, SD = 14.04), followed by dermatology (JSE M = 106.64, SD = 16.90), obstetrics and gynecology (JSE M = 106.48, SD = 14.31), and pediatrics (JSE M = 106.02, SD = 12.18). Lower JSE scores were frequently found in procedure-oriented departments, including ophthalmology (JSE M = 96.00, SD = 11.99), orthopedics (JSE M = 99.21, SD = 14.34), radiology (JSE M = 99.35, SD = 13.69), anesthesiology (JSE M = 100.16, SD = 14.25), and urology (JSE M = 101.18, SD = 15.15). Except for general medicine, the top medical specialties with the highest JSE scores (dermatology, obstetrics and gynecology, and pediatrics) also had significantly higher percentages of women physicians. Conversely, orthopedics and surgery had significantly lower percentages of women physicians. Anesthesiology was characterized by a high percentage of women (47.0%) but lower JSE scores (M = 103.61, SD = 15.0) than specialty areas.

**Table 2:** Jefferson scale of empathy mean scores and proportion of women according to future specialty among Japanese resident physicians.

	n	Mean	SD	Women (%)	p-value
General medicine	50	109.74	14.04	28.0%	0.996
Dermatology	31	106.64	16.90	51.6%	**0.003**
Obstetrics and gynecology	48	106.48	14.31	58.3%	** < 0.001**
Pediatrics	64	106.02	12.18	39.1%	0.041
Psychiatry	65	105.23	16.81	30.8%	0.609
Rehabilitation*	14	105.17	16.41	28.6%	1.0
Emergency medicine	60	104.86	14.41	28.3%	0.957
Internal medicine	325	104.60	14.56	28.0%	0.986
Pathology*	10	103.80	11.54	20.0%	0.734
Otorhinolaryngology*	20	103.66	11.43	50.0%	**0.027**
Others*	19	103.44	15.92	31.6%	0.797
Surgery	90	103.39	15.32	16.7%	**0.011**
Plastic surgery*	17	102.35	17.88	29.4%	1.0
Neurosurgery*	21	102.28	11.87	23.8%	0.808
Clinical laboratory*	1	102.00	n/a	0.0%	1.0
Not yet decided	55	101.26	13.65	30.9%	0.623
Urology	30	101.18	15.15	20.0%	0.318
Anesthesiology	49	100.16	14.25	46.9%	**0.002**
Radiology*	20	99.35	13.69	20.0%	0.615
Orthopedics	51	99.21	14.34	15.7%	**0.043**
Ophthalmology*	17	96.00	11.99	29.4%	1.000

*Fisher’s exact test for proportion of women doctors; otherwise, the chi-squared test was used. Bold font indicates statistically significant differences.

Table 3 compares future general medicine candidates with those of other specialties. The results showed no significant differences in hospital-level variables. However, future general medicine candidates were less likely to include PGY-2 residents (40.0%, p = 0.020) and less likely to have a mentor (46.0%, p = 0.028). In addition, they had significantly higher GM-ITE scores and significantly higher JSE scores than residents of other specialties (general medicine JSE M = 109.74, SD = 14.04; other specialty M = 103.2, SD = 14.96; effect size Cohen’s d = 0.436, p = 0.003).

**Table 3:** Comparison of future general medicine candidates with the rest of the specialties.

	Future general medicine candidate, n = 50	Others, n = 774	p-value
Hospital-level variables			
Hospital type (%)			0.6481
University	5 (10.00)	93 (12.02)	
University branch	2 (4.00)	53 (6.85)	
Community	43 (86.00)	628 (81.14)	
Hospital location (%)			0.421
Urban	12 (24.00)	227 (29.33)	
Rural	38 (76.00)	547 (70.67)	
Number of beds	537.3 (241.1)	553.3 (217.4)	0.6148
Resident-level variables			
Sex (%)			0.9956
Men	36 (72.00)	557 (71.96)	
Women	14 (28.00)	217 (28.04)	
PGY (%)			**0.0201**
PGY-1	30 (60.00)	334 (43.15)	
PGY-2	20 (40.00)	440 (56.85)	
ED duties per month (%)			0.8295
None	1 (2.00)	36 (4.65)	
1–2	8 (16.00)	122 (15.76)	
3–5	36 (72.00)	551 (71.19)	
6 or more	5 (10.00)	65 (8.40)	
Average number of inpatients in charge (%)			0.6316
0–4	17 (34.00)	203 (26.23)	
5–9	26 (52.00)	471 (60.85)	
10–14	5 (10.00)	73 (9.43)	
15 or more	2 (4.00)	27 (3.49)	
Resident duty hours per week (%)			0.6132
59 or fewer	15 (30.00)	283 (36.56)	
60–79	23 (46.00)	310 (40.05)	
80 or more	12 (24.00)	181 (23.39)	
Have mentor(s)			**0.0275**
Yes	23 (46.00)	534 (68.99)	
No	27 (54.00)	240 (31.01)	
JSE	109.7 (14.04)	103.2 (14.96)	**0.0028**
GM-ITE score	48.54 (7.02)	46.14 (7.31)	**0.0243**

*PGY* postgraduate years, *GM*-*ITE* general medicine in-training examination, *ED* emergency department.

Bold font indicates statistically significant differences.

Finally, multiple linear regression analyses were performed using all the above hospital-level and resident-level variables to determine whether they were relevant factors for the JSE scores (Table 4). The VIF for all items ranged from 1.04 to 1.48, with a mean of 1.14. Among these, only the coefficients found for women ($\beta$ = 2.42, 95% CI 0.10–4.7328; p = 0.041) and future general medicine candidates ($\beta$ = 6.68, 95% CI 2.39–10.9; p = 0.002) were statistically significant. Finally, a sensitivity analysis was performed, testing various in- and out-of-specialty and item variables. However, only the above variables for women and general medicine were significant, while the other variables, including resident- and hospital-level factors, were not significantly linked with JSE scores.

**Table 4:** Multivariate linear regression analysis for the Jefferson scale of empathy.

	Coefficient	95% CI		p-value
		Lower	Upper	
Hospital-level variables				
Hospital type (%)				
University	0 (reference)	n/a	n/a	n/a
University branch	− 0.9265	− 7.3645	5.5115	0.7775
Community	− 2.4905	− 7.1342	2.1531	0.2925
Hospital location (%)				
Urban	0 (reference)	n/a	n/a	n/a
Rural	− 0.6059	− 3.0063	2.0175	0.6455
Number of beds	0.000663	− 0.0055	0.00681	0.8322
Resident-level variables				
Sex (%)				
Men	0 (reference)	n/a	n/a	n/a
Women	2.4176	0.1025	4.7328	**0.0407**
PGY (%)				
PGY-1	0 (reference)			
PGY-2	0.5362	− 1.5695	2.642	0.617
ED duties per month (%)				
None	0 (reference)	n/a	n/a	n/a
1–2	2.9464	− 2.6921	8.5848	0.305
3–5	0.8515	− 4.599	6.302	0.759
6 or more	1.8245	− 4.6625	8.3116	0.5807
Average number of inpatients in charge (%)				
0–4	0 (reference)	n/a	n/a	n/a
5–9	0.664	− 1.8483	3.1762	0.6038
10–14	− 0.08924	− 4.1744	3.9959	0.9658
15 or more	− 0.2595	− 6.3496	5.8305	0.9333
Resident duty hours per week (%)				
59 or fewer	0 (reference)	n/a	n/a	n/a
60–79	1.3471	− 1.0903	3.7845	0.278
80 or more	1.5356	− 1.3346	4.4059	0.2936
Have mentor(s)?				
Yes	0 (reference)	n/a	n/a	n/a
No	− 0.8496	− 3.1144	1.4152	0.4614
Future specialties				
Others	0 (reference)	n/a	n/a	n/a
Future general medicine candidate	6.6773	2.3859	10.9686	**0.0024**
GM-ITE score	0.1258	− 0.02153	0.2732	0.094

*PGY* postgraduate years, *GM*-*ITE* general medicine in-training examination, *ED* emergency department.

To adjust for potential confounders of medically significant factors associated with the Jefferson Scale of Empathy, the following variables were incorporated in the multivariate analysis: hospital-level variables (hospital type, hospital location, number of beds) and resident-level variables (sex, postgraduate year, ED duties per month, average number of inpatients in charge, resident duty hours per week, study time per week, presence of a mentor(s), future general medicine candidates, and GM-ITE scores).

Bold font indicates statistically significant differences.

## Discussion

This study used a nationwide cross-sectional survey across Japan to assess variations in empathy levels among residents (PGY-1 and PGY-2) based on their intended future specialty. The findings, measured using the JSE, revealed disparities in empathy levels correlated with future specialty choices before residents’ specialty training, with the highest levels observed in those leaning toward the choice of general medicine. In addition, after adjusting for the training environment and resident level, general medicine was associated with significantly higher empathy scores among the 19 primary medical specialties as well as with a higher number of women majoring in the field. The Cronbach’s alpha of the JSE was high for medical students (0.80) and physicians (0.80) in the US (0.84), which is comparable to the level reported for medical students (0.80) and physicians (0.81) in Japan; this study demonstrated similar results (0.84)^20,21^. Our discussion focuses on three areas to help frame our results: (1) differences in empathy by specialty, (2) potential explanations for high empathy levels among general medicine candidates, and (3) potential explanations for high empathy among women.

### Differences in empathy among each specialty

Previous research indicates that physicians employed in patient-centered specialties, such as general medicine, internal medicine, psychiatry, and pediatrics, exhibit notably higher levels of empathy compared to those in procedure- and technology-centered specialties, such as surgery, anesthesiology, plastic surgery, orthopedics, and neurosurgery^3,21,32^. The findings of a Polish study are particularly noteworthy as it highlights that family physicians display the highest levels of empathy, which aligns with the current study’s observation of high empathy among Japanese generalists^32^. Although not directly comparable numerically to our study, as shown in Table 2, the empathy of residents who sought to train in procedure- and technology-oriented departments tended to be lower. This trend was similar to previous studies^16,22,24^. Other studies have suggested that a patient-centered communication style predicts medical error outcomes in primary care physicians but not in surgeons, proceduralists, and technique-centered practice, and the reasons are still unknown^33^. It remains unclear whether these disparate levels of empathy are due to self-selection into a specialty or the result of their unique training effects and experiences^17,24,34,35^. In this study, the evidence highlights the differences in empathy levels depending on the specialty of interest that exist even before residents begin specialty training. Several other studies have suggested that empathy may decrease after surgical training^23,24^. This may be due to the unique empathic characteristics of surgeons, which differ from those in general medicine and internal medicine^24^. Therefore, some training for personnel in specialties where empathy tends to be low may be helpful, and numerous educational studies have been reported^19,20,36,37^.

### Potential explanations for high empathy levels among general medicine physicians

Several previous studies have consistently demonstrated that physicians practicing in environments that prioritize patient-centered principles exhibit elevated empathy levels^3,21^. Notably, the prospective general medicine physicians in our study displayed significantly higher empathy levels than the average residents. Since they were residents before embarking on their specialized training in general medicine, it is improbable that the influence of their major program accounts for this difference. Instead, it is plausible that residents who initially chose to pursue a career in general medicine may have prioritized empathy as a crucial factor in their decision-making process. Thus, we must carefully consider the potential impact of the general internal medicine specialty certification program, which commenced in 2018, and its stated competencies^38^, as it may further illuminate the observed higher empathy levels among general internists. The General Medicine Board-Certified Programs include human-centered medicine and care (patient-centered medicine, family-oriented medicine and care, and communication to facilitate collaboration with patients and families) as the first of six competencies^25,38,39^. That is, residents who agree with the importance of these competencies are likely to further increase their empathy scores by applying them to general medicine. In Japan, there is only a slight disparity in salaries among medical specialties, and the lack of competition allows residents to choose their specialty according to their medical interests and aptitude^21^. While this situation may be unique and different from that in North America^40^, the apparent high level of empathy among residents who wish to pursue general medicine adds new evidence to previous studies.

### Potential explanations for high empathy among women

Numerous studies have consistently suggested a higher level of empathy among women physicians and medical students^3,17,21,41,42^. This finding has historically been attributed to intrinsic factors (e.g. biological and evolutionary sex differences) and extrinsic factors (e.g. socialization, sex [gender] role norms, and societal expectations)^21,42^. In our study of medical residents, the average JSE scores were higher among women, which is consistent with previous studies. At least one study from Japan also noted that among women residents, the medical specialty choice is influenced by work-life integration (e.g. perceived balance between work and childcare)^21^. However, we were unable to correlate the choice of specialty among women physicians with levels of empathy found among physicians in various specialties, primarily because of the relatively low representation of women among the residents in our study.

This study has additional limitations. First, as this was a cross-sectional study, we cannot know whether PGY-1 residents will, in fact, enter their identified future specialty as they reported in our questionnaire. The percentage of PGY-2 residents that aspired to become general medicine physicians was slightly lower compared to PGY-1 residents. However, the data from PGY-2 residents are more reliable because the career paths of almost all residents are already determined at the time of the end-of-year examinations. Second, we excluded respondents that chose more than one specialty. The results would likely have been slightly different if they had been included. Third, general medicine in Japan is a relatively new specialty, and the fields of general internal medicine, hospital medicine, and family medicine overlap; hence, some residents of internal medicine might pursue hospital medicine or general internal medicine. Fourth, the current distribution of training facilities in Japan consists of approximately 45% university hospitals and 55% city hospitals. Notably, this study’s data are significantly well represented by the participation of the city hospitals. This can be attributed to the pronounced popularity of the GM-ITE among city hospitals, contributing approximately 80% of the participants, despite the involvement of over 630 training facilities annually. However, while there exists a disparity in the participant ratio between university hospitals and city hospitals, the ratio of affiliations between examinees and training participants remains nearly identical. Fifth, this study lacks specific details on the training departments chosen by each resident. The Japanese postgraduate clinical training requirements entail a minimum of 24 weeks in internal medicine, 12 weeks in emergency medicine, and 4 weeks each in surgery, pediatrics, obstetrics and gynecology, psychiatry, and community medicine. However, it is essential to consider that if the residents receive additional training in certain technology-oriented departments during the selection period, such as surgery or anesthesiology, it could potentially impact empathy levels and, consequently, lead to different study results^24^.

Our survey is the first to be used nationally to reveal differences in empathy among Japanese medical residents according to their future specialties. Our study confirms a high degree of empathy among physicians who aspire to be general medicine physicians, a specialty that values patient-centeredness. Empathy tended to be higher in more human-centered departments and lower in more procedure- and technology-oriented departments. However, in multivariate analysis, only aspirations for general medicine and being a woman were linked with significantly higher levels of empathy. No differences were found after adjustment for other medical specialties, training, working environment, or other factors. The findings of this study strengthen the evidence from previous studies conducted outside of Japan. Our results may have implications of postgraduate education; for example, empathy training strategies may be created for use in specialties wherein physicians tend to have lower empathy scores. However, further research is needed to determine why there are differences in the levels of empathy among applicants and to what extent these differences affect clinical practice.

## Data Availability

The data supporting the findings of this study are available from the corresponding author, T. W, General Medicine Center, Shimane University Hospital (e-mail. shimanegp@gmail.com), upon reasonable request.
